# The right ventricle shows distinct wall motion characteristics in L-TGA vs. D-TGA/atrial switch

**DOI:** 10.1186/1532-429X-11-S1-P28

**Published:** 2009-01-28

**Authors:** Michelle Savacool, Philip Kilner, David Sahn, Willem Helbing, Harold Litt, Emanuela Valsangiacomo, Florence Sheehan

**Affiliations:** 1 University of Washington, Seattle, WA USA; 2 Royal Brompton Hospital, London, UK; 3 Oregon Health & Science University, Portland, OR USA; 4 Erasmus Medical Center, Rotterdam, Netherlands; 5 University of Pennsylvania, Philadelphia, PA USA; 6 University Children's Hospital, Zurich, Switzerland

**Keywords:** Wall Motion, Right Ventricle, Atrial Switch, Develop Heart Failure, Right Ventricle Function

## Background

Patients whose right ventricles (RV) support the systemic load may develop heart failure. However it is unknown whether the RV remodels similarly in different etiologies of systemic RV.

## Methods

RV wall motion and shape were measured from 3D reconstructions generated from magnetic resonance images in 25 patients with transposition of the great arteries after atrial switch (D-TGA), 17 patients with L-TGA and 9 normal subjects. Wall motion was measured in 11 regions of the RV free wall and septum and normalized by dividing by the square root of body surface area. Regional RV Shape was measured as eccentricity (range from 0 for a line to 1 for a circle) in 20 short-axis slices of the RV.

## Results

**Figure 1:**
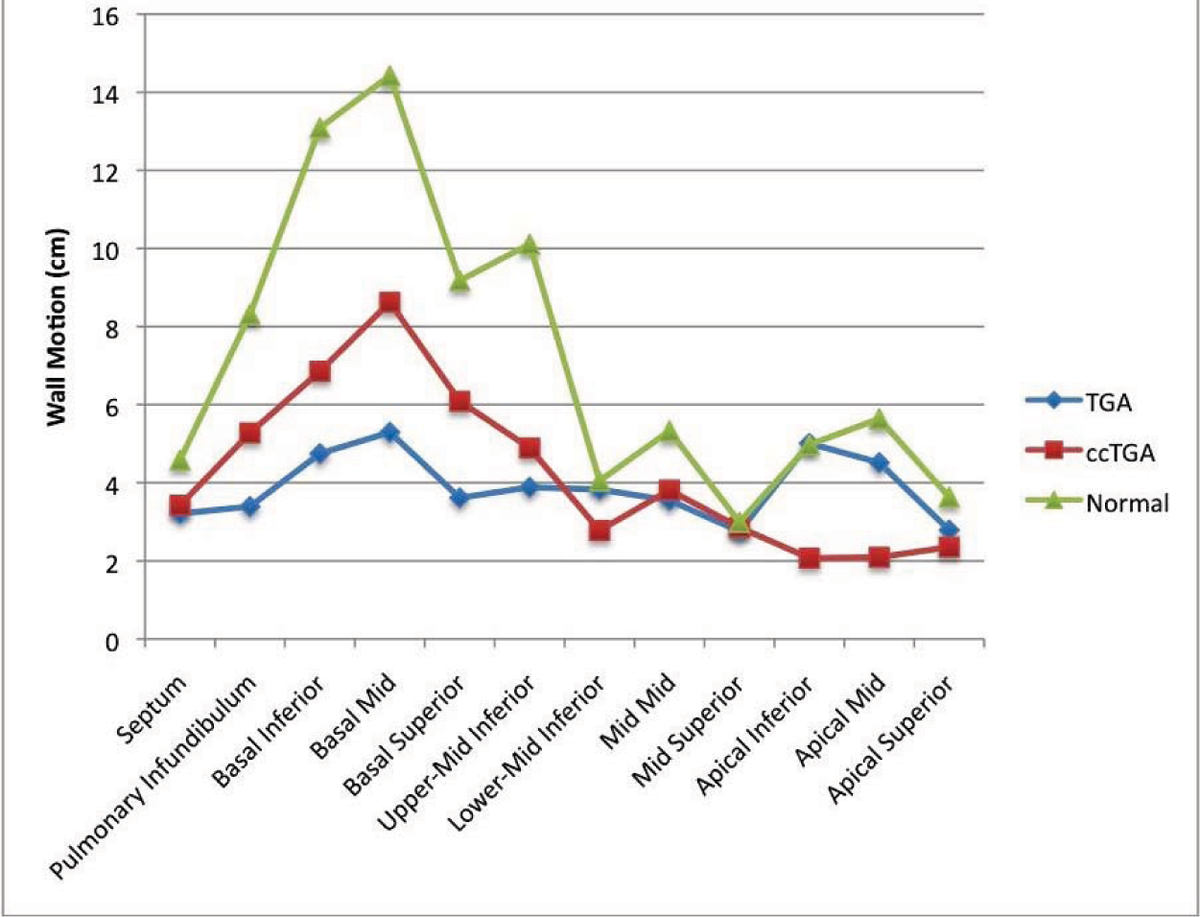
**Regional wall motion (cm)**.

Systemic RV patients had severe RV dilatation (end diastolic volume index 137 ± 37 vs. 84 ± 22 ml/m^2^ in normals, p < .001), poor RV function (ejection fraction (EF) 32 ± 7 vs. 55 ± 5%, p < .001), and more circular cross sections (eccentricity .83 to .93 vs. below .66 for normals, p < .001 for all slices). L-TGA patients had better RV function than D-TGA patients (EF 35 ± 7 vs. 30 ± 7%, p < .05). Both groups had significantly depressed wall motion in most regions compared to normal. However L-TGA had better wall motion at the base than D-TGA but contracted more poorly at the apex. Figure 1.

## Conclusion

Systemic RVs are dilated, spherical, and poorly functioning. L-TGA and D-TGA systemic RVs have distinctive wall motion and remodeling patterns. The functional and morphological response to systemic pressure in the RV may differ depending on the anomaly.

